# Questioning flecainide's mechanism of action in the treatment of catecholaminergic polymorphic ventricular tachycardia

**DOI:** 10.1113/JP272497

**Published:** 2016-10-31

**Authors:** Alan J. Williams, Mark L. Bannister, N. Lowri Thomas, Markus B. Sikkel, Saptarshi Mukherjee, Chloe Maxwell, Kenneth T. MacLeod, Christopher H. George

**Affiliations:** ^1^ School of MedicineCardiff UniversityCardiffCF14 4XNUK; ^2^ Myocardial Function SectionNational Heart and Lung InstituteImperial College LondonLondonW12 0NNUK

Flecainide attenuates cardiac Ca^2+^ cycling abnormalities in malignant catecholamine‐triggered arrhythmias but its mechanism of action remains highly contentious. We read with interest the study of Yang *et al*. (2016) that used *in silico* predictions in an attempt to determine the relative contribution of block by flecainide of ryanodine receptor 2 (RyR2) and the Na^+^ channel to the overall therapeutic effect of this drug in catecholaminergic polymorphic ventricular tachycardia (CPVT). Flecainide's actions were modelled by considering its inhibition of the Na^+^ current (*I*_Na_) and RyR2 in combination and individually. The authors conclude that the effects of the drug on Na^+^ channels are insufficient to explain its efficacy in CPVT, while its block of RyR2 alone was as effective as the combined block of *I*_Na_ and RyR2.

Our concerns about the usefulness of this approach are twofold:

## (1) Purported block of RyR2 by flecainide

The blocking parameters used in Yang *et al*. (2016) are based on values reported in Hilliard *et al*. (2010) and subsequent publications from the same group. The major problem with the use of these parameters to model open channel block by flecainide of RyR2‐mediated Ca^2+^ release from the sarcoplasmic reticulum (SR) is that Hilliard *et al*. (2010) did not demonstrate block of this current, rather they reported partial block of K^+^ movement in the opposite (cytosol to luminal) direction.

To fully understand the problem it is necessary to outline some basic features of RyR2 channel structure and function.

**Figure 1:**
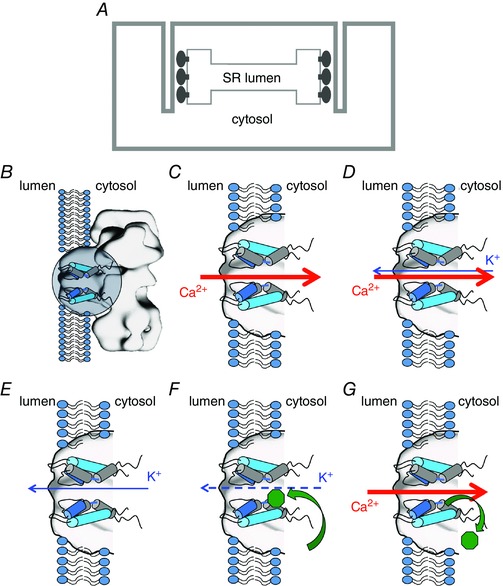
**Ionic fluxes and interaction of flecainide in RyR2** *A*, schematic representation of the location and orientation of RyR2 in the cardiac myocyte SR membrane system. The trans‐membrane domain contains the pore‐forming region (PFR) and the large cytosolic domain abuts transverse tubule invaginations of the sarcolemma. *B–G*, schematic diagrams depicting the open RyR2 PFR (*B*, indicated by circle) together with relevant cation fluxes and interactions of flecainide (green octagon). Only two of the four PFR monomers are shown for clarity. *C*, to initiate contraction Ca^2+^ is released from the SR, flowing down its concentration gradient through the open RyR2 PFR. *D*, to dissipate the diffusion potential generated by Ca^2+^ release, a counter current of K^+^ flows from the cytosol to the SR lumen, at least in part through RyR2. *E*, the RyR2 blocking parameters used in Yang *et al*. (2016) are based on data reported in Hilliard *et al*. (2010) that describe the effects of flecainide on the cytosolic to luminal flux of K^+^. *F*, under these conditions flecainide enters the cytosolic cavity of RyR2 and partially occludes the pore. *G*, the physiologically relevant flux of Ca^2+^ from the lumen to the cytosol displaces flecainide (Bannister *et al*. 2015). As a consequence open channel block of RyR2 by flecainide can play no part in the effectiveness of this drug in the treatment of CPVT.

RyR2 provides a ligand‐regulated pathway for the release of Ca^2+^ from the cardiac SR to initiate myocyte contraction. In its open conformation Ca^2+^ flows through the pore‐forming region (PFR) of RyR2, down its concentration gradient, from the SR lumen to the cytosol. The key structural features of the RyR2 PFR, based on recent high‐resolution cryo‐electron microscopy investigations (Yan *et al*. 2014), are shown schematically in Fig. 1. Ca^2+^ leaving the SR enters the luminal mouth of the open PFR before passing through a region equivalent to the selectivity filter of the more discriminating monovalent‐selective channels and eventually into a large, water‐filled, cytosolic cavity contiguous with the bulk cytosol, lined with trans‐membrane helices (Fig. 1 *C*). RyR2‐mediated Ca^2+^ release is an electrogenic process during which a charge‐compensating K^+^ counter current flows from the cytosol into the SR, at least in part, through RyR2 (Fig. 1 *D*).

To monitor flecainide's actions on RyR2 Hilliard *et al*. (2010) incorporated channels into phospholipid bilayers and measured K^+^ flux under voltage clamp conditions. RyR2 incorporates into the bilayer in a fixed orientation so that its configuration is defined. In these investigations, the net direction of K^+^ flux through RyR2 was from the cytosolic to luminal side of the channel, driven by a trans‐membrane potential with the solution at the cytosolic side held at +40 mV relative to that at the luminal side of the channel (Fig. 1 *E*). Flecainide was added to the cytosolic solution and, from here, interacts with RyR2 (Fig. 1 *F*). When bound, flecainide partially occludes the RyR2 PFR and individual blocking events are resolved with dwell‐times in the millisecond range. A residual current of ∼20% of the full open current continues to flow with flecainide bound. The key point of our comment on the work of Yang *et al*. (2016) is that the experiments of Hilliard *et al*.(2010) merely demonstrate block of cytosolic to luminal flux of K^+^ through RyR2; they provide no information on the ability of flecainide to block the flux of cations, and in particular Ca^2+^, in the physiologically relevant direction through RyR2, that is, from the SR lumen to the cytosol. For this reason the use of parameters of block determined by Hilliard *et al*. (2010) in the models described in Yang *et al*. (2016) is inappropriate and irrelevant and their conclusion that the action of flecainide on RyR2 is sufficient to explain flecainide efficacy in CPVT, is unsound.

The possibility that block by flecainide could influence RyR2‐mediated Ca^2+^ release was examined in Bannister *et al*. (2015) using individual recombinant human RyR2 channels in planar bilayers. In these investigations we demonstrated that flecainide limits cytosolic to luminal K^+^ flux through RyR2 by interacting at a site within the cytosolic cavity of the PFR. Flecainide only has access to this site from the cytosol and, crucially, the affinity with which flecainide is bound is insufficient to prevent it being displaced by cation flux in the physiologically relevant, luminal to cytosolic, direction. This is the case for either K^+^ flux driven by a trans‐membrane potential of −40 mV or by Ca^2+^ flux driven by a luminal to cytosolic concentration gradient (the flux equivalent to Ca^2+^ release from the SR) (Fig. 1 *G*). Further, flecainide did not influence RyR2 gating and had negligible effect on the charge‐compensating K^+^ current carried collectively by RyR2 and the SR K^+^ channel during Ca^2+^ release. Based on these observations we concluded that the efficacy of flecainide in the treatment of CPVT is not the result of a direct interaction with RyR2. Given this, we believe that the inclusion of parameters describing block of RyR2 by flecainide in the models used in Yang *et al*. (2016) is unjustified and will inevitably lead to spurious conclusions.

## (2) Block of Na^+^ channel by flecainide

A key factor in the conclusion of Yang *et al*. (2016) that block of the Na^+^ channel alone is insufficient to explain flecainide efficacy in CPVT was provided by modelling flecainide's action in the absence of RyR2 block. However, by their own admission, the IC_50_ used in the model to describe flecainide's inhibition of Ca^2+^ waves ‘does not separate out external contributions to this parameter, such as lowered junctional Ca^2+^ brought about by *I*_NCX_ activity via lowered [Na^+^]’ resulting from block of *I*_Na_, as proposed by Sikkel *et al*. (2013). It would be very interesting to see if inclusion of this potential mechanism of action of flecainide in the model could provide an alternative explanation for the efficacy of flecainide in CPVT.

## Additional information

### Competing interests

None declared.

### Funding

Work described in this letter was funded by the British Heart Foundation (PG14/34/30835).
